# Tonin Overexpression in Mice Diminishes Sympathetic Autonomic Modulation and Alters Angiotensin Type 1 Receptor Response

**DOI:** 10.3389/fmed.2018.00365

**Published:** 2019-01-23

**Authors:** Zaira Palomino Jara, Marcelo Yudi Icimoto, Rodrigo Yokota, Amanda Aparecida Ribeiro, Fernando dos Santos, Leandro Ezequiel de Souza, Ingrid Kazue Mizuno Watanabe, Maria do Carmo Franco, Jorge Luiz Pesquero, Maria Claudia Irigoyen, Dulce Elena Casarini

**Affiliations:** ^1^Disciplina de Nefrologia, Departamento de Medicina, Universidade Federal de São Paulo, São Paulo, Brazil; ^2^Cleveland Clinic, Cleveland, OH, United States; ^3^Departmento de Biofisica, Escola Paulista de Medicina, Universidade Federal de São Paulo, São Paulo, Brazil; ^4^Divisao de Hipertensao, Escola de Medicina, Instituto do Coracao, Universidade de São Paulo, São Paulo, Brazil; ^5^Departamento de Biofisica, Universidade Federal de Minas Gerais, Belo Horizonte, Brazil

**Keywords:** tonin, serino protease, AT1R, losartan, renin angiotensin system, autonomic control, angiotensin II, angiotensin 1-7

## Abstract

**Background:** Tonin, a serine-protease that forms Angiotensin II (AngII) from angiotensinogen, is increased in failing human heart samples. Increased blood pressure (BP) and decreased heart rate (HR) variabilities are associated with higher risk of cardiovascular morbidity. Losartan has been used to reduce hypertension and, therefore, lowers the risk of fatal and non-fatal cardiovascular events. Determination of tonin's impact on BP and HR variabilities as well as the impact of losartan remain questions to be elucidated.

**Aim:** Evaluation of cardiovascular autonomic profile in transgenic mice overexpressing the rat tonin enzyme TGM'(rton) and the impact of AT1 receptor blocker, losartan.

**Methods:** Male C57BL/6 (WT) and TGM'(rTon) mice were cannulated for recording BP (Windaq, 4 MHz) for 30 min at baseline and 30 min after losartan injection (20 mg/kg). BP and HR variabilities were analyzed in time and frequency domain method. Low-frequency (LF) and high-frequency (HF) components were identified for sympathetic and parasympathetic modulations analysis. Ang I, AngII, and Ang1-7 were quantified by high performance liquid chromatography method. The total enzymatic activity for AngI, AngII, and Ang1-7 formation was evaluated in the heart and plasma by Liquid chromatography mass spectrometry (LC-MS/MS).

**Results:** At the baseline TGM'(rTon) exhibited higher BP, lower cardiac LF, higher cardiac HF, lower LF/HF, and lower alpha index than wild type (WT). After losartan injection, TGM'(rTon) mice presented an additional decrease in cardiac LF and increase in HF in relation to baseline and WT. In the vasculature, losartan caused decreased in BP and LF of systolic BP in WT mice in relation to its baseline. A similar effect was observed in the BP of TGM'(rTon) mice; however, LF of systolic BP increased compared to baseline. Our data also indicates that AT1R receptor signaling has been altered in TGM’(rTon)mice. Interestingly, the dynamics of the renin-angiotensin system kinetics change, favoring production of Ang1-7.

**Conclusion:** Autonomic evaluation of TGM’(rTon) mice indicates an unclear prognosis for diseases that affect the heart. HR variability in TGM’(rTon) mice indicates high risk of morbidity, and sympathetic and parasympathetic modulation indicate low risk of morbidity. The low risk of morbidity could be the biased production of Ang1-7 in the heart and circulation; however, the altered response of AT1R in the TGM’(rTon) remains to be elucidated, as well aswhether that signaling is pro-protection or pro-pathology.

## Introduction

Angiotensin II (Ang II), the main agonist of the renin-angiotensin system, plays an important role in the maintenance of a healthy heart. Inotropism and chronotropism are a few of its direct roles in the heart (1–3), and as part of the integrated system it participates in hemodynamic stability (4). Deleterious effects of overstimulation by Ang II contribute to an array of cardiovascular diseases. More specifically, this hormone is implicated in a chain of events that contribute to the initiation and progression of cardiovascular disease. Ang II has been implicated in risk factors such hypertension, hyperlipidemia, and diabetes, in development processes such as atherosclerosis and left ventricular dysfunction, leading to ventricular hypertrophy and fibrosis followed by congestive heart failure. Thus, Ang II is a key target for the treatment of cardiovascular diseases (3, 5, 6). This powerful peptide can be formed by many enzymatic pathways within the renin-angiotensin system, including renin and angiotensin converting enzyme (ACE), via tonin, cathepsin, and chymase (7–9). However, the main routes to formation of Ang II are tissue specific. A systematic study reported that 80% of the total AngII-forming activity in heart was inhibited by serine protease inhibitor (10). This observation explained production of Ang II in heart failure patients treated with ACE inhibitors. Tonin is a serine protease that can form Ang II from Ang I and directly from angiotensinogen (Agt) (11–13). Therefore, this enzyme could be another source of Ang-II, independent of that produced via ACE. We have advanced only a few steps toward a more precise definition of the role of tonin-angiotensin II in the puzzling system of angiotensin II-generating enzymes. This lack of clarity may be due to the unique characteristics of that differ from other converting enzyme(s). A closer relationship between tonin-angiotensin II system and norepinephrine (14–16) system could be part of an integrated system by which myriad of complex mechanisms maintains homeostasis of the cardiovascular autonomic system. Taken together, tonin, a serine protease could play a role in the development of the cardiovascular diseases. Thus, our goal is to evaluate how the upregulation of tonin affects the risk of cardiovascular morbidity, its impact on the other angiotensins and on the main receptor of the renin-angiotensin system, the angiotensin type 1 receptor (AT1R).

## Materials and Methods

### Mice

Wild type C57BL/6 (WT) and homozygous transgenic mice overexpressing the rat tonin enzyme [TGM'(rton)] were used in this study. Mice were housed in a temperature-controlled room (21 ± 1°C) under a 12:12-h dark light cycle, received standard laboratory chow and water *ad libitum*. The research ethics committee of the Federal University of São Paulo approved experimental protocols used in this study.

### Generation of Transgenic Mice

Transgenic TGM'(rTon) mice were generated by microinjection of the rat tonin transgene into zygotes as described by Ribeiro et al. (17, 18). TGM'(rTon) mice exhibited higher tonin activity in the brain, heart, kidney, liver, and bladder compared to WT (17).

### Catheter Implantation and Hemodynamic Recording

Catheters filled with heparinized saline were implanted into carotid artery and jugular vein of the anesthetized mice (80 mg/kg ketamine and 12 mg/kg xylazine, i.p.) Animals were placed in individual clean cages with free access to food and water. A heating lamp was used to keep the animals warm until their full recovery. Forty-eight hours later if a mouse presented signs of decreased activity, reluctance to move, hunched posture, and ungroomed fur, it was withdrawn from the study. The animals that recovered well were connected to computerized acquisition systems by connecting the arterial cannula to an electromagnetic transducer (Kent Instruments) by a polyethylene tube. This transducer was linked to an amplifier (General Purpose Amplifier-Stemtech, Inc.) and connected to a computer with an analog to digital converter board (Windaq/DataQ). The venous catheter was tethered to a 25 cm polyethylene tube filled with heparinized saline. To allow for stabilization of their blood pressure, the animals remained connected for at least half an hour to the system. After the adaptation period, 30 min of real-time blood pressure and heart rate signals were recorded before and after a single injection of losartan (20 mg/Kg, iv) in conscious animals who were free to move. Losartan was injected through the venous catheter.

### Heart Rate and Blood Pressure Variability

Analysis of blood pressure signals was performed using an algorithm implemented in acquisition system Windaq/DataQ. This software allowed the detection of the maximal value in the blood pressure curve, beat to beat, providing the values of systolic blood pressure (SBP). Heart rate was determined from the pulse interval (PI) between two systolic peaks. Heart rate variability was measured by linear methods in time and frequency domains. Temporal series of PI and SBP (30 min of baseline recording) were analyzed in time domain, obtaining the total variance of PI (VAR PI) and the variance of SBP (VAR SBP). Mean square root of differences between consecutives PI (RMSSD) was also evaluated. For the frequency domain, one spectrum was obtained for each segment and the oscillatory components were quantified in two different frequencies: low frequency (LF) from 0.1 to 1 Hz and high frequency (HF) from 1 to 5 Hz. The results are represented by absolute values (ms^2^ and mmHg^2^), the percentage of total spectrum (%) and normalized units (nu) (percentage of LF and HF bands only).

### Sample Collection

Animals were euthanized by decapitation at 12–14 weeks old. Blood samples were collected and plasma was immediately separated from the whole blood by centrifugation at 1,500 rpm for 10 min and stored at −80°C. Hearts were rapidly removed, snap frozen in liquid nitrogen and stored at −80°C until experimental procedures were performed.

### Sample Preparation and Quantification of Angiotensins by High Performance Liquid Chromatography

Atria and right and left ventricles were dissected for homogenization in 100 mM sodium phosphate, 240 mM sucrose, 300 mM NaCl, and the protease inhibitors: 1 mM EDTA (metalloprotease inhibitor), 1 μM o-phenanthroline (metalloprotease inhibitor), 120 μM pepstatin A (aspartyl protease inhibitor), 1 μM PMSF (serine protease inhibitor), and 1 mM 4-chloromercuribenzoic acid (cysteine protease inhibitor) at a ratio of 5 ml of buffer per gram of tissue. Homogenates were centrifuged at 20,000x g for 1 h at 4°C. In each resulting supernatant, 1 nmol of Sar^1^-AngII was introduced as an internal standard. Samples were purified and concentrated using C18 Sep-Pak columns previously activated with methanol (5 ml), tetrahydrofuran (5 ml), hexane (5 ml), methanol (5 ml), and water (10 ml). After loading the samples into the column, purification was performed by washing the column with ultrapure water. Samples were eluted from the column using 5 ml of ethanol/acetic acid/water in the proportion (90:4:6). The eluates were lyophilized then rehydrated in 500 ul of mobile phase A (5% acetonitrile in 0.1% orthophosphoric acid. Each sample was filtered (0.22 um pore size filter) and injected into the HPLC. The peptides were separated on the reverse phase column Aquapore 300 ODS (250 x 4.6 mm 7 μ) connected to a high-performance liquid chromatography system (Shimatzu) with 214 nm UV detection. The peptide separation was performed with 20 min of linear gradient from 5 to 35% mobile phase B (95% acetonitrile in 0.1% orthophosphoric acid) after 5 min of isocratic gradient over, at constant flow rate of 1.5 ml/min. Sar^1^-AngII, AngI, AngII, and Ang1-7 synthetic peptides (Sigma) were used as standards and the angiotensins in each sample were identified according to the angiotensin standards retention time. The concentrations were expressed in picomol per gram of tissue.

### Sample Preparation for Enzymatic Activity

Hearts were dissected into atria, right ventricle, and left ventricle. These chambers were homogenized in 50 mM Tris/HCl, 0.5 mM ZnCl_2_ buffer, pH 7.5, followed by centrifugation at 10,000 rpm at 4°C for 10 min. The resulting supernatants were separated from the pellet. The protein concentration of the supernatants was determined by Bradford (Biorad) method as recommended by the manufacture. Protein quantification was performed on the supernatants for further normalization of each peptide chromatographic-peak-area.

### Total Angiotensin II and Angiotensin 1-7 Forming Enzymes Activities

Tetradecapeptide (TDP) (10 nmol), a synthetic analog of angiotensinogen, was incubated in 1 ml of buffer (50 mM Tris/HCl, 0.5 mM ZnCl_2_, pH 7.5) containing either 50 ul of plasma, 100 ul of atria or right or left ventricle homogenates at 37°C for 24 h. Aliquots were collected at 10, 20, 40, 60, 120, 240, 360, and 1,440 min into a tube containing 10% formic acid. Angiotensin I, Angiotensin II, and Angiotensin 1-7 were quantified by liquid chromatography/mass spectrometry.

#### Chromatographic Conditions

LC experiments were carried out with an Agilent1290 System with a Luna C18 100 × 2 mm, 3 μm column at 40°C with a 9 min gradient of eluent water and acetonitrile with 0.1% formic acid at a flow rate of 400 μL/min. The injection volume was set to 5 μL.

#### Mass Spectrometry Conditions

The AB SCIEX QTRAP® 5500 system was operated with Turbo V Electrospray Ionization probe. The 3 angiotensins were confirmed by comparison with angiotensin standard retention time, 3 MRM transitions per peptide to allow quantitation and identification based on the ratio of quantifier and qualifier transitions and an exclusive scan mode, the Enhanced Product Ion (EPI) which is at least 100 times more intense than a common MS/MS scan. The method used triggers an ion trap MS/MS scan on the ion of interest once the MRM intensity exceeds a certain threshold. The MS/MS data obtained confirms the identity of the angiotensins with the entire MS/MS spectrum rather than just with the presence of two diagnostic fragments.

### Statistics

All data are expressed as mean and standard error of mean. Significance level was determined by using two-way ANOVA with Bonferroni *post-hoc* test or with multiple *t*-test comparison. 95% confidence of interval was used and *P*-value < 0.05 was considered statistically significant.

## Results

### Tonin Enzyme Overexpression *in vivo* Increased Blood Pressure

**Figure 1:**
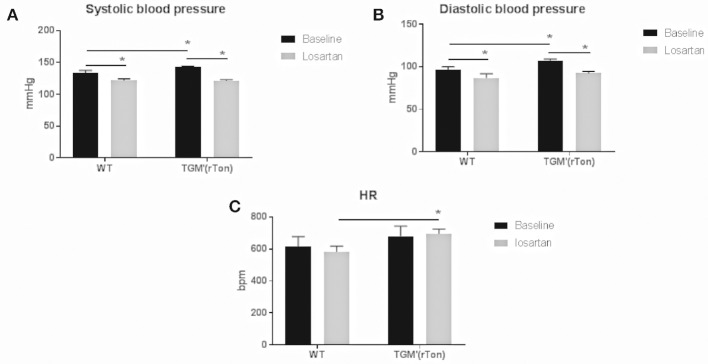
Hemodynamic evaluation in wild type (WT) and transgenic mice overexpressing rat tonin [TGM'(rTon)] at baseline and after angiotensin type 1 receptor blocker, losartan: Overexpression of tonin enzyme increased the systolic **(A)** and diastolic **(B)** blood pressure (BP, *p* = 0.0464) without significant changes in the heart rate (HR, **C)**. Blockage of AT1R reduced systolic and diastolic BP in both strains (*p* = 0.0036). Mice given losartan maintained similar HR to baseline. *N* = 4, Two-way ANOVA ^*^p < 0.05, followed by bonferroni *post hoc*, bar graphs are means ±SEM.

Overexpression of tonin caused an increase in diastolic and systolic BP (*p* = 0.0464) without altering heart rate. Pharmacological blockade of AT1R reduced systolic and diastolic BP in both strains (*p* = 0.0036; Figure 1). However, when the delta value of BP was evaluated before and after losartan injection, it was observed that losartan induced greater reduction of BP in TGM'(rTon) than WT (21.46 vs. 11. 86 mmHg, respectively).

### Losartan, Angiotensin Type 1 Receptor Blocker, Further Decreases Sympathetic and Increases Parasympathetic Modulation on the Cardiac Autonomic Control in TGM'(rTon) Mice

**Figure 2:**
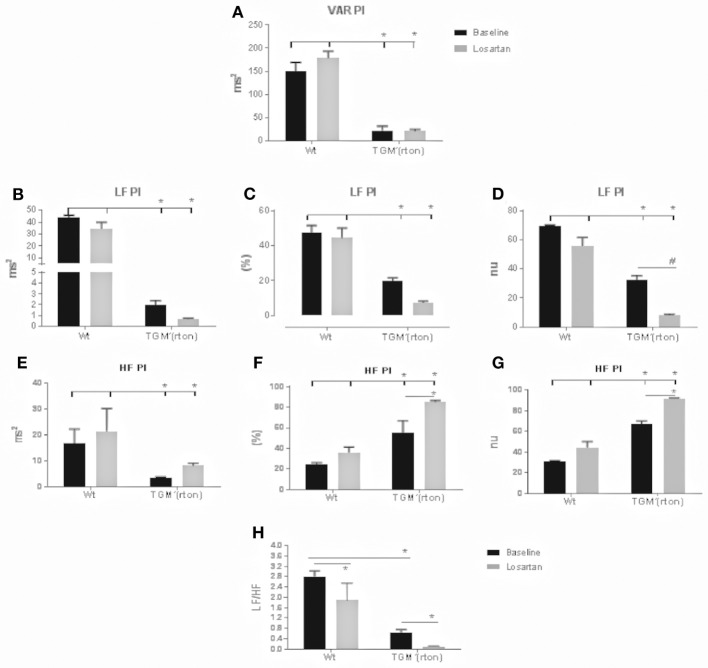
Time and frequency domain evaluation of heart rate (HR) variability in wild type (WT) and transgenic mice overexpressing rat tonin [TGM'(rTon)] at baseline and after angiotensin type 1 receptor blocker, losartan: Variance of pulse interval (VAR PI) **(A)** low frequency band of pulse interval (LF PI), including absolute **(B)**, percent **(C)**, and normalized units **(D)**, high frequency of pulse interval (HF PI) including absolute **(E)**, percent **(F)**, and normalized units **(G)**, sympathovagal balance (LF/HF) **(H)**. LF bands represent the sympathetic modulation whereas HF parasympathetic. *N* = 4, Two-way ANOVA followed by bonferroni *post hoc* ^*^p < 0.05, bar graphs are means ±SEM.

At baseline the TGM'(rTon) mice showed a reduction in total HR variability, represented by variance of pulse interval (VAR PI). Together a clear reduction in the sympathetic specific spectrum band (LF) was observed when analyzed the total (ms^2^), percentage (%), and normalized (nu). Interestingly, an elevation of parasympathetic modulation was observed when analyzing its specific spectrum band (HF). Consequently, a decreased sympathovagal balance was observed when compared to WT group (Figure 2). In the TGM'(rTon), blockade of AT1R caused further decrease and increase in the sympathetic and parasympathetic modulation, respectively. Both effects were greater in the TGM'(rTon) when compared to the WT group.

### Losartan, Angiotensin Type 1 Receptor Blocker, did not Affect Blood Pressure Variability Even Though the Sympathetic Modulation of the Vasculature Was Altered in TGM'(rTon) Mice

**Figure 3:**
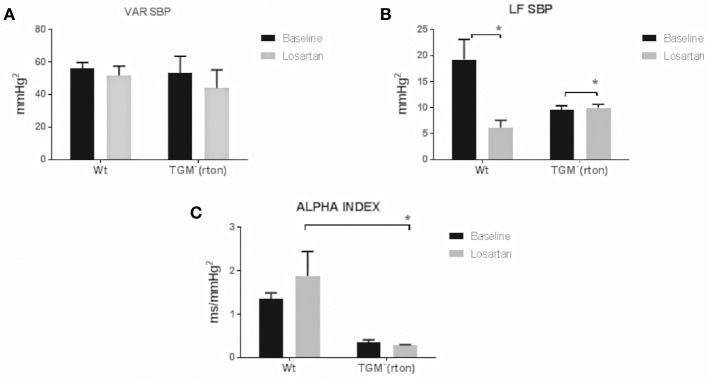
Time and frequency domain evaluation of blood pressure variability in wild type (WT) and transgenic mice overexpressing rat tonin [TGM'(rTon)] at baseline and after angiotensin type 1 receptor blocker, losartan: Variance of SBP (VAR SBP) **(A)**, low frequency band of SBP (LF SBP) **(B)** and alpha index **(C)**. LF bands represent sympathetic modulation and alpha index represents baroreflex sensitivity. *N* = 4, Two-way ANOVA followed by bonferroni *post hoc* ^*^p < 0.05, bar graphs are means ±SEM.

TGM'(rTon) at baseline and after angiotensin receptor blockage did not significantly alter the systolic BP variability represented by VAR SBP. Pharmacological blockade of AT1R caused reduction of sympathetic modulation in the controls, but it increased in the TGM'(rTon) (Figure 3). After losartan injection, TGM'(rTon) exhibited lower baroreflex sensitivity measured by the alpha index compared to WT group.

### Tonin Overexpression Alters the AngI, AngII, and Ang1-7 Content in the Heart

**Figure 4:**
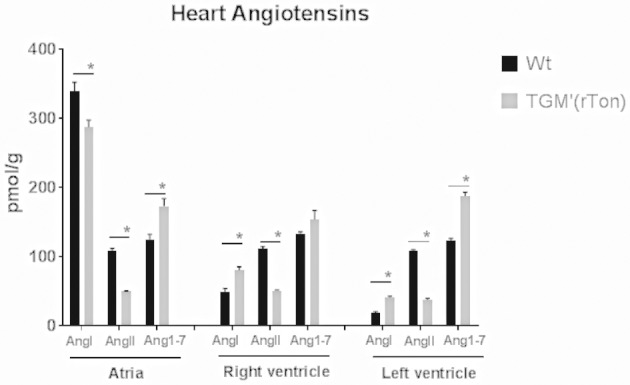
Angiotensin content in three chambers of the heart in wild type (WT) and transgenic mice overexpressing rat tonin [TGM'(rTon)]: Heart angiotensin concentration of WT and TGM'(rTon) mice were quantified by High Performance Liquid Chromatography. AngI, AngII, and Ang1-7 were measured in each chamber. *N* = 4, Two-way ANOVA followed by multiple *t*-tests ^*^p < 0.05, bar graphs are means ±SEM.

Within each WT and TGM'(rTon) group, levels of AngII and Ang1-7 did not differ among heart compartments, indicating that the steady-state levels of AngII and Ang1-7 levels follow similar regulation. Levels of Ang I in the atria were 2 times higher than in the right and left ventricle in WT group and 1 time higher in TGM'(rTon). When levels of angiotensins are compared between groups, TGM'(rTon) mice showed decreased levels of AngI in atria and higher levels of AngI in both right ventricle and left ventricle. Contrastingly, AngII levels were uniformly decreased in TGM'(rTon) heart chambers compared with WT group (Figure 4). This observation was accompanied by a large increase in Ang1-7. These results indicate changes in angiotensin processing, resulting in production of Ang1-7 instead of production of Ang II in TGM'(rTon) mice.

### Tonin Overexpression Alters the Angiotensins Formation Dynamic in the Heart

**Figure 5:**
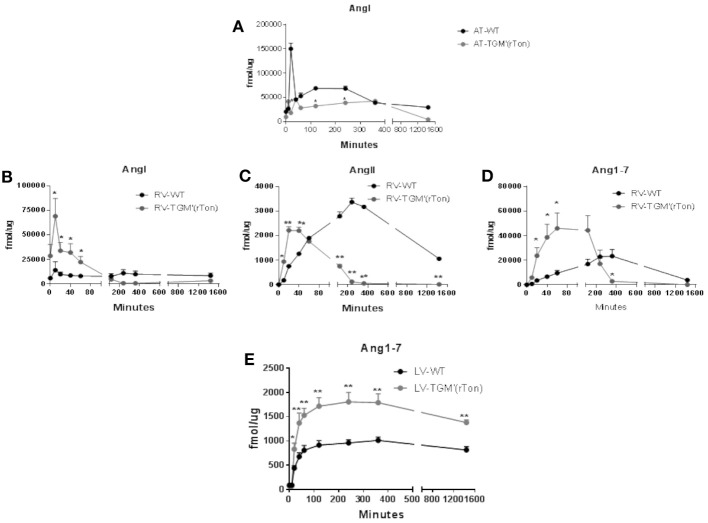
Total AngI, AngII, and Ang1-7 formation activity in atria, right ventricle and left ventricle of wild type (WT) and transgenic mice overexpressing rat tonin [TGM'(rTon)]: AngI levels in the atria after incubation with tetradecapeptide as substrate at 0, 10, 20, 40, 60, 120, 240, 360, and 1,440 min (*n* = 2) **(A)**. AngI **(B)**, AngII **(C)**, and Ang1-7 **(D)** in the right ventricle (RV) after incubation with tetradecapeptide as substrate at 0, 10, 20, 40, 60, 120, 240, 360, and 1,440 min by LC-MS/MS (*n* = 4). Ang1-7 **(E)** levels in the left ventricle (LV) after incubation with AngII as substrate at 0, 10, 20, 40, 60, 120, 240, 360, and 1,440 min by LC-MS/MS (*n* = 4). Multiple *t*-tests were performed, ^*^p < 0.05, ^**^p < 0.001 time points are represented by means ± SEM.

The proteolytic capacity of each compartment generating angiotensins were tested *in vitro* by a mass spectrometry-based assay. Extracts of atria, right ventricle and left ventricle of both WT and TGM'(rTon) mice were preincubated with tetradecapeptide and levels of Ang I, Ang II, and Ang1-7 were quantified by LC-MSMS in a time-dependent manner. Total atria extract were capable of rapidly consuming the TDP, generating Ang I in the first minute of incubation until a maximum concentration was reached between the 120 and 240 min for WT group and 360 min for TGM'(rTon) mice (Figure 5A). Corroborating the observation made using HPLC assay, atrial production of Ang I was shown to be higher in WT than TGM'(rTon)mice.

Levels of angiotensins generated by right ventricle extracts from tetradecapetide precursor were also measured. There was a rapid formation (0–40 min) of Ang I, AngII, and Ang1-7 in the TGM'(rTon) group compared to the WT group (120–360 min), contrasting with the delay in formation observed in the WT extracts. Interestingly, the highest peak of Ang I production corresponds to 20 min in TGM'(rTon) group; in contrast, the highest peak of Ang I formation was reached at 4 h in the WT mice (Figure 5B). A similar difference was obtained for Ang II (40 min for TGM'(rTon) group vs. 4 h for WT group; Figure 5C) and for Ang1-7 (60 min for TGM'(rTon) and 360 min for WT group, Figure 5D). It is noteworthy that the total formation of Ang1-7 was 2 times higher than WT group. These data suggest that RV extracts of TGM'(rTon) produce all angiotensins faster than WT mice.

The levels of Ang1-7 were also measured using left ventricle extracts using Ang II as precursor (Figure 5E). The levels of Ang1-7 was significantly higher for TGM'(rTon) compared to WT mice. Total Ang1-7 formation in the atria, RV and LV corroborate the observation of Ang1-7 content by HPLC, i.e., higher levels of Ang1-7. Similarly, total AngII formation in the RV corroborate the observation found by HLPC, lower levels of AngII in the heart.

### Tonin Impacts the Circulating Renin Angiotensin System Homeostasis

**Figure 6:**
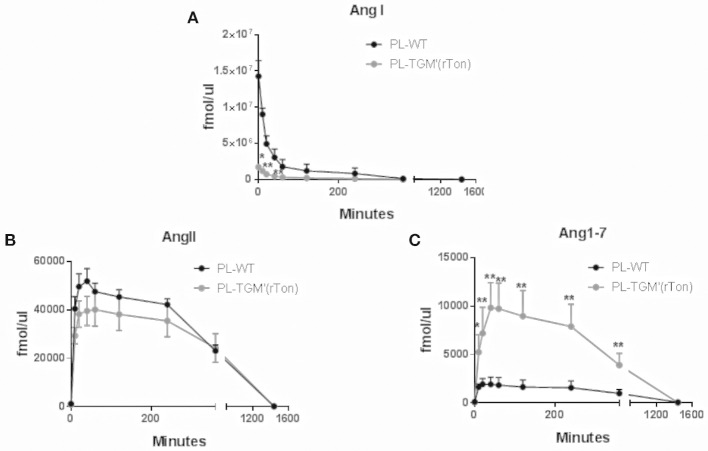
Total Ang I, Ang II, and Ang1-7 formation in the plasma of wild type (WT) and transgenic mice overexpressing rat tonin [TGM'(rTon)]: AngI **(A)**, AngII **(B)**, and Ang1-7 **(C)** in the plasma (PL) after incubation with tetradecapeptide as substrate at 0, 10, 20, 40, 60, 120, 240, 360, and 1,440 min by LC-MS/MS (*n* = 4). Multiple *t*-test were performed ^*^p < 0.05, time points are represented by means ±SEM.

The proteolytic capacity of plasmatic proteases generating angiotensins from tetradecapeptide were also analyzed. Plasmatic proteases were capable of rapidly consuming tetradecapeptide, generating high concentrations of Ang I in the first 10 min of incubation for both TGM'(rton) and WT mice (Figure 6A). However, the amounts of plasmatic Ang I produced by TGM'(rTon) group was 10 times lower than the amounts obtained by WT mice, but still higher than the amounts obtained using atrial and RV extracts. The amounts of AngII formed in the plasma from TDP as substrate were similar in both mice (Figure 6B). Again, the amount of Ang1-7 was higher in the TGM'(rTon) compared to WT group (Figure 6C).

**Figure 7:**
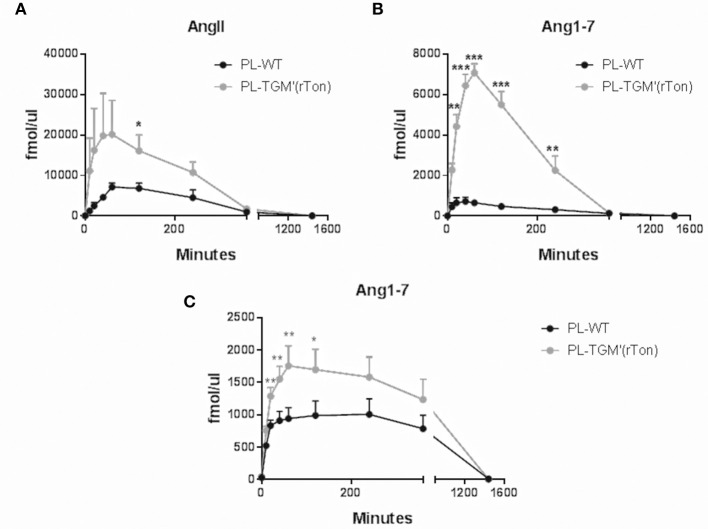
Total Ang II and Ang1-7 formation in the plasma of wild type (WT) and transgenic mice overexpressing rat tonin [TGM'(rTon)]: AngII **(A)** and Ang1-7 **(B)** in the plasma (PL) after incubation with AngI as substrate at 0, 10, 20, 40, 60, 120, 240, 360, and 1,440 min by LC-MS/MS (*n* = 4). Ang1-7 **(C)** in the plasma after incubation with AngII (*n* = 4–5). Multiple *t*-test were performed ^*^p < 0.05, time points are represented by means ± SEM.

The amounts of Ang II and Ang1-7 generated by plasmatic proteases using Ang I and Ang II as precursors were also measured. When the tested precursor was Ang I (Figures 7A,B), the level of Ang II and Ang 1-7 levels were significantly higher in TGM'(rTon) compared to control mice, reaching a maximum concentration after 40–60 min of incubation. Again, higher Ang 1-7 levels were obtained using Ang II as precursor in the TGM'(rTon) group when compared to the WT mice (Figure 7C).

## Discussion

In the present study TGM'(rTon) mice exhibited an increase in BP at pre-hypertensive levels; similar results were published by Cardoso et al. (19). One of the mechanisms underlying this phenotype is an increase in circulatory concentrations of Ang II, which was two-fold higher than the increased concentration of Ang 1-7 observed. However, it is worth noting that when we evaluated the proteolytic capacity of the plasmatic proteases to the tetradecapeptide, a synthetic analog of angiotensinogen which mimics the peptide initiator of the renin angiotensin cascade, similar levels of Ang II were produced when compared to WT group. Only when incubated with Ang I was it possible to see increased levels of Ang II. This finding indicates that in circulation, the slight increase of AngII levels observed in the TGM'(rTon) mice are derived from Ang I only. Tonin synthesized in the brain most likely is being released into the circulation. Once released into the circulation, tonin is immediately trapped by α1-Macroglobulin (α1-M) to form a tonin-α1-M (20). Hence, previous attempts to detect tonin activity in plasma (human and rat) were unsuccessful (21–24). Only using the immunoimmobilization technique was it possible to detect this enzyme in the blood (20). The tonin-α1-M complex forms Ang II solely from Ang I and not from angiotensinogen, due a lack of fit in the catalytic site now partly occupied by the inhibitor. Ikeda et al. (20) observed that when tonin was intravenously administered as a bolus injection, tonin's fate followed two paths. First, fast clearance of 125 I-labeled tonin from the circulation was observed. Secondly, when tonin is in complex with-α1-M, Ang II-forming activity by solid-phase antibody-bound tonin-α1-M complex persisted for over 1 h and even showed an increase after rapid clearance in the early phase. The formation of Ang II was demonstrated despite the complete inhibition of converting enzyme (25).

Blockade of AT1R by losartan resulted in greater BP reduction in the TGM'(rTon) group than the in the WT group; this observation indicates that AT1R signaling was preponderant in the acute control of BP in TGM'(rTon) group. Taken together, increased Ang II due to increased circulating pathway of Ang I-tonin-AngII-AT1R is one of the mechanisms of a net result of higher BP in the TGM'(rTon) mice; however, we cannot be certain that the only route for higher Ang II formation in the circulation is via tonin in the TGM'(rTon) mouse, because tonin can affect the expression of other enzymes such as ACE as we have previously shown (17).

When evaluating the autonomic control of the heart, we found contradictory observations. On the one hand, reduction of HR variability indicates risk for developing cardiovascular morbidity associated with decreased baroreflex sensitivity (alpha index). HR variability is an indirect estimator of autonomic modulation of heart rate and is considered a risk marker in critical illness, particularly in heart failure, and severe sepsis. A reduced HR variability has been found in critically ill patients and has been associated with neuro-autonomic uncoupling or decreased baroreflex sensitivity. On the other hand, reduction of sympathetic modulation together with increase in parasympathetic modulation indicates a cardioprotective state. Increase in sympathetic activity has been positively associated with hypertension in humans and animal models (26, 27). Furthermore, acute injection of tonin *in vivo* caused imbalance of the autonomic control reflected by an increase in the sympathetic tone (28). The question remains: why do TGM'(rTon) mice decrease sympathetic modulation with higher Ang II plasmatic environment? We hypothesize that this surprising observation could be explained by a defective tonin-AngII-catecholamines axis produced in a chronic tonin mileu. Interestingly, parasympathetic tone was increased when we evaluated autonomic control of the heart. This observation can be explained by the increased levels of Ang 1-7 in the heart and in the circulation. The parasympathetic excitatory effect of Ang 1-7 was consistently reported in the literature (29, 30). After losartan injection, sympathetic decrease and parasympathetic increase was observed in both groups; however, statistical significance is only seen in the TGM'(rTon) mice. This finding indicates that AT1R control of the autonomic function in the heart was maintained or even exacerbated. In other words, this observation again indicates that AT1R signaling is predominant in the TGM'(rTon) mice compared to WT group. Sensitization of AT1R could be one of the mechanisms to increase responsiveness of AT1R. Surprisingly, the Ang II levels in the TGM'(rTon) heart were lower compared to the WT group, even though increased tonin activity was observed in the atria and left ventricle as we previously described (18). The lower Ang II levels could be the result of changes in the dynamics of the renin-angiotensin system kinetics, favoring the formation of Ang 1-7 from Ang II. Therefore, Ang II formation is rapidly broken down to Ang1-7. This observation is consistent with our previous report, where we observed increased ACE2 expression in a mileu of increased tonin activity. Thus, it seems that super activation of ACE2, an Ang1-7 forming enzyme, overrides activation of tonin and other Ang II forming enzymes. This pathway could be one of the mechanisms for a decrease in AngII and an increase in Ang1-7 (31, 32).

When we evaluated the autonomic control of the vasculature neither tonin nor losartan had an impact on the BP variability in both groups. As for the sympathetic modulation at baseline, the TGM'(rTon) group showed similar sympathetic modulation to what was observed in the WT group. Furthermore, in contrast to what we observed in the autonomic modulation of the heart, the TGM'(rTon) group responded to AT1R blockade in a opposite fashion than the WT group; in other words, the AT1R was not as responsive as in the WT group for the autonomic control of the vasculature. This observation could be explained by the observation of AT1R desensitization by Cardoso et al. (19). In their work, injection of Ang II was not able to increase BP in TGM(rTon) when compared to WT. Therefore, a desensitized AT1R was overridden by the other receptors that work in a opposite manner to control the autonomic function of the vasculature; thus a net result of increase sympathetic modulation was observed in the TGM'(rTon).

In summary, TGM'(rTon) mice presented indicators of lower risk for vascular morbidity compared to WT mice. However, our findings presented an unclear prognosis for diseases that affect the heart. HR variability in TGM'(rTon) mice indicates high risk of morbidity, and sympathetic and parasympathetic modulation indicate low risk of morbidity. One of the underlying mechanisms for the protective prognosis may rely on the Ang1-7 effect. AT1R signaling properties have changed in different fashion in the heart compared to the vasculature in the TGM'(rTon) mice. This observation needs further study as well as understanding the mechanism of impairment that may have occurred in the tonin-AngII-catecholamines axis.

## Ethics Statement

This study was carried out in accordance with the recommendations of UNIFESP CEDEME Committee of Ethics in Research. The protocol was approved by the CEP committee of Ethics in Research.

## Author Contributions

ZJ, RY, IW, AR, and LS performed experiments. ZJ, FdS, MYI, and MF evaluated data. ZJ and MYI wrote the manuscript. DC, MCI, JP, and ZJ conducted the study. All authors read and approved the final version of the manuscript.

### Conflict of Interest Statement

The authors declare that the research was conducted in the absence of any commercial or financial relationships that could be construed as a potential conflict of interest. The reviewer HU declared a non-overlapping institutional affiliation, though no other collaboration, with one of the authors, ZJ to the handling Editor.

## References

[1] De Mello WC. Intracrine action of angiotensin II in the intact ventricle of the failing heart: angiotensin II changes cardiac excitability from within. Mol Cell Biochem. (2011) 358:309–15. 10.1007/s11010-011-0981-4 21744071 PMC3779357

[2] De Mello WC. Novel aspects of angiotensin II action in the heart. Implications to myocardial ischemia and heart failure. Regul Pept. (2011) 166:9–14. 10.1016/j.regpep.2010.10.003 20934462

[3] Ferrario CM Mullick AE. Renin angiotensin aldosterone inhibition in the treatment of cardiovascular disease. Pharmacol Res. (2017) 125(Pt A):57–71. 10.1016/j.phrs.2017.05.020 28571891 PMC5648016

[4] Sapouckey SA Deng G Sigmund CD Grobe JL. Potential mechanisms of hypothalamic renin-angiotensin system activation by leptin and DOCA-salt for the control of resting metabolism. Physiol Genomics (2017) 49:722–32. 10.1152/physiolgenomics.00087.2017 28986397 PMC5814669

[5] Ferrario CM Strawn WB. Role of the renin-angiotensin-aldosterone system and proinflammatory mediators in cardiovascular disease. Am J Cardiol. (2006) 98:121–8. 10.1016/j.amjcard.2006.01.059 16784934

[6] Zhang ZM Wang BX Ou WS Lv YH Li MM Miao Z. Administration of losartan improves aortic arterial stiffness and reduces the occurrence of acute coronary syndrome in aged patients with essential hypertension. J Cell Biochem. (2018). 10.1002/jcb.27856. [Epub ahead of print]. 30362602

[7] Belova LA. Angiotensin II-generating enzymes. Biochemistry (2000) 65:1337–45. 11173502 10.1023/a:1002848402911

[8] Carl-McGrath S Grantzdorffer I Lendeckel U Ebert MP Rocken C. Angiotensin II-generating enzymes, angiotensin-converting enzyme (ACE) and mast cell chymase (CMA1), in gastric inflammation may be regulated by *H. pylori* and associated cytokines. Pathology (2009) 41:419–27. 10.1080/00313020902885037 19424904

[9] Ahmad S Ferrario CM. Chymase inhibitors for the treatment of cardiac diseases: a patent review (2010-2018). Expert Opin Ther Pat. (2018) 28:755–64. 10.1080/13543776.2018.1531848 30278800 PMC6240413

[10] Urata H Healy B Stewart RW Bumpus FM Husain A. Angiotensin II-forming pathways in normal and failing human hearts. Circ Res. (1990) 66:883–90. 10.1161/01.RES.66.4.883 2156635

[11] Schiller PW Demassieux S Boucher R. Substrate specificity of tonin from rat submaxillary gland. Circ Res. (1976) 39:629–32. 10.1161/01.RES.39.5.629 184974

[12] Grise C Boucher R Thibault G Genest J Formation of angiotensin II by tonin from partially purified human angiotensinogen. Can J Biochem. (1981) 59:250–5. 10.1139/o81-034 6265047

[13] Pesquero JL Boschcov P Oliveira MC Paiva AC. Effect of substrate size on tonin activity. Biochem Biophys Res Commun. (1982) 108:1441–6. 10.1016/S0006-291X(82)80068-5 6295383

[14] Garcia R Boucher R Genest J. Tonin activity in rat saliva: effect of sympathomimetic and parasympathomimetic drugs. Can J Physiol Pharmacol. (1976) 54:443–5. 10.1139/y76-063 974872

[15] Garcia R Kondo K Scholkens B Boucher R Genest J. Effect in vivo of beta-adrenergic stimulation, angiotensin II, dibutyryl cyclic AMP, and theophylline on tonin concentration in rat saliva and submaxillary gland. Can J Physiol Pharmacol. (1977) 55:983–9. 10.1139/y77-135 200323

[16] Gutkowska J Thibault G Cantin M Garcia R Genest J. Kallikrein concentration in submandibular glands of rats chronically treated with isoproterenol. Can J Physiol Pharmacol. (1983) 61:449–56. 10.1139/y83-069 6554083

[17] Ribeiro AA Palomino Z Lima MP Souza LE Ferreira DS Casarini DE. Characterization of the renal renin-angiotensin system in transgenic mice that express rat tonin. J Renin Angiotensin Aldosterone Syst. (2015) 16:947–55. 10.1177/1470320315595572 26216430

[18] Ribeiro AA Amorim RP Palomino ZJ de Paula Lima M Moraes-Silva IC Souza LE. (Pro)renin receptor expression in myocardial infarction in transgenic mice expressing rat tonin. Int J Biol Macromol. (2018) 108:817–25. 10.1016/j.ijbiomac.2017.10.179 29102794

[19] Cardoso CC Alenina N Ferreira AJ Qadri FLima MP Bader M. Increased blood pressure and water intake in transgenic mice expressing rat tonin in the brain. Biol Chem. (2010) 391:435–41. 10.1515/bc.2010.040 20180641

[20] Ikeda M Sasaguri M Maruta H Arakawa K. Formation of angiotensin II by tonin-inhibitor complex. Hypertension (1988) 11:63–70. 10.1161/01.HYP.11.1.63 2448241

[21] Boucher R Saidi M Genest J A new angiotensin converting enzyme system. Hypertension (1972) 1972:512–23.

[22] Boucher R Asselin J Genest J A new enzyme leading to the direct formation of angiotensin II. Circ Res. (1974) 35:I-203–I-212.

[23] Boucher R Demassieux S Garcia R Genest J. Tonin, angiotensin II system. A review. Circ Res. (1977) 41(4 Suppl 2):26–9. 10.1161/01.RES.41.4.26 20244

[24] Boucher R Garcia R Gutkowska J Demassieux S Genest J. Tonin–angiotensin II system in hypertension. Clin Sci Mol Med. (1978) (Suppl 4):183s−6s. 10.1042/cs055183s 215373

[25] Nussberger J Brunner DB Waeber B Brunner HR. Specific measurement of angiotensin metabolites and *in vitro* generated angiotensin II in plasma. Hypertension (1986) 8:476–82. 10.1161/01.HYP.8.6.476 3011664

[26] Papaioannou VE Verkerk AO Amin AS de Bakker JM. Intracardiac origin of heart rate variability, pacemaker funny current and their possible association with critical illness. Curr Cardiol Rev. (2013) 9:82–96. 10.2174/157340313805076359 22920474 PMC3584310

[27] Jandackova VK Scholes S Britton A Steptoe A. Are changes in heart rate variability in middle-aged and older people normative or caused by pathological conditions? Findings from a large population-based longitudinal cohort study. J Am Heart Assoc (2016) 5:2. 10.1161/JAHA.115.002365 26873682 PMC4802439

[28] Damasceno DD Lima MP Motta DF Ferreira AJ Quintao-Junior JF Pesquero JL. Cardiovascular and eletrocardiographic parameters after tonin administration in Wistar rats. Regul Pept. (2013) 181:30–6. 10.1016/j.regpep.2012.12.009 23318501

[29] Nautiyal M Shaltout H de Lima Ado Nascimento DC Chappell KM Diz DI. Central angiotensin-(1-7) improves vagal function independent of blood pressure in hypertensive (mRen2)27 rats. Hypertension (2012) 60:1257–65. 10.1161/HYPERTENSIONAHA.112.196782 23045456 PMC3491885

[30] Dartora DR Irigoyen MC Casali KR Moraes-Silva IC Bertagnolli M Santos RAS. Improved cardiovascular autonomic modulation in transgenic rats expressing an Ang-(1-7)-producing fusion protein. Can J Physiol Pharmacol. (2017) 95:993–8. 10.1139/cjpp-2016-0557 28459154

[31] Soler MJ Batlle M Riera M Campos B Ortiz-Perez JT Perez-Villa F. ACE2 and ACE in acute and chronic rejection after human heart transplantation. Int J Cardiol. (2018) 275:59–64. 10.1016/j.ijcard.2018.10.002 30314840

[32] Yu J Wu Y Zhang Y Zhang L Ma Q Luo X. Role of ACE2-Ang (1-7)-Mas receptor axis in heart failure with preserved ejection fraction with hypertension. Zhong Nan Da Xue Xue Bao Yi Xue Ban (2018) 43:738–46. 10.11817/j.issn.1672-7347.2018.07.007 30124209

